# Cyanobacteria and Algae-Derived Bioactive Metabolites as Antiviral Agents: Evidence, Mode of Action, and Scope for Further Expansion; A Comprehensive Review in Light of the SARS-CoV-2 Outbreak

**DOI:** 10.3390/antiox11020354

**Published:** 2022-02-10

**Authors:** Biswajita Pradhan, Rabindra Nayak, Srimanta Patra, Prajna Paramita Bhuyan, Soumya Ranjan Dash, Jang-Seu Ki, Siba Prasad Adhikary, Andrea Ragusa, Mrutyunjay Jena

**Affiliations:** 1 Algal Biotechnology and Molecular Systematic Laboratory, Post Graduate Department of Botany, Berhampur University, Bhanja Bihar, Berhampur 760007, India; pradhan.biswajita2014@gmail.com (B.P.); rabindran335@gmail.com (R.N.); srdash.sanu@gmail.com (S.R.D.); 2 Department of Biotechnology, Sangmyung University, Seoul 03016, Korea; kijs@smu.ac.kr; 3 Cancer and Cell Death Laboratory, Department of Life Science, National Institute of Technology, Rourkela 769008, India; 518LS2007@nitrkl.ac.in; 4 Department of Botany, Maharaja Sriram Chandra Bhanja Deo University, Baripada 757003, India; prajnabhuyan2017@gmail.com; 5 Department of Biotechnology, Institute of Science, Visva-Bharati, Santiniketan 731235, India; adhikarysp@gmail.com; 6 Department of Biological and Environmental Sciences and Technologies, Campus Ecotekne, University of Salento, Via Monteroni, 73100 Lecce, Italy; 7 CNR-Nanotec, Institute of Nanotechnology, Via Monteroni, 73100 Lecce, Italy

**Keywords:** antiviral drug, bioactive metabolites, coronaviruses, SARS-CoV-2, COVID-19, immunomodulation, marine algae

## Abstract

COVID-19—a severe acute respiratory syndrome disease caused by coronavirus 2 (SARS-CoV-2)—has recently attracted global attention, due to its devastating impact, to the point of being declared a pandemic. The search for new natural therapeutic drugs is mandatory, as the screening of already-known antiviral drugs so far has led to poor results. Several species of marine algae have been reported as sources of bioactive metabolites with potential antiviral and immunomodulatory activities, among others. Some of these bioactive metabolites might be able to act as antimicrobial drugs and also against viral infections by inhibiting their replication. Moreover, they could also trigger immunity against viral infection in humans and could be used as protective agents against COVID-In this context, this article reviews the main antiviral activities of bioactive metabolites from marine algae and their potential exploitation as anti-SARS-CoV-2 drugs.

## 1. Introduction

The World Health Organization (WHO) confirmed the emergence of a novel coronavirus (nCoV-2019, later defined SARS-CoV-2) on 12 January 2020 in Wuhan, China and named the derived pathology “coronavirus disease 2019 (COVID-19)”, an acute respiratory tract infection [1]. COVID-19 cases quickly spread around the world, and it was declared a pandemic on March 11, 2020 [2]. Cough, fever, headache, sore throat, dyspnea, and weariness are common COVID-19 symptoms which can lead to a severe respiratory infection, pneumonia, and multiple organ failure, resulting in patient death. COVID-19 is particularly life-threatening to people with diabetes, cardiovascular disease, hypertension, cancer, HIV, and a variety of autoimmune diseases [3].

In order to rapidly find a treatment for COVID-19, many already-known synthetic drugs have been tested against SARS-CoV-2 although mostly with low or questionable efficacy and a variety of adverse effects. In this regard, the screening of compounds extracted from natural sources could yield new structures able to interact strongly with the virus, thus inhibiting it. The marine ecosystem is the most biodiverse ecosystem [4] and it can provide a variety of bioactive metabolites. Algae-derived bioactive metabolites have been shown to have several potential therapeutic effects, including antiviral activity [5,6]. For example, bioactive metabolites derived from marine algae have been tested against human cytomegalovirus (HCMV), human enterovirus, influenza virus, human immunodeficiency virus type-1 (HIV-1), herpes simplex virus (HSV), hepatitis B virus, murine norovirus, and respiratory syncytial virus (RSV) and displayed strong antiviral activity [7,8]. Thus, algal metabolites might also have a therapeutic effect by inhibiting SARS-CoV-2, halting illness progression.

Immunity is crucial for the treatment of viral diseases such as COVID-19 [9]. Antiviral immunity has been proven in studies against a variety of viral pathologies, and algal metabolites have been shown to possess promising activity [8]. To date, many metabolites from plants and animals, as well as marine species and microbes, have been tested against HIV and HSV [10]. Thus, the use of immune system-boosting algae-derived bioactive metabolites may play a leading role in fighting coronavirus infections by improving innate immune responses. Nutraceuticals from *Spirulina* have been extensively studied and made commercially available as an innate enhancement [11]. Furthermore, their antiviral activity against HIV and HSV was also evaluated both in vitro and in vivo [12].

Although some vaccines against COVID-19 have been produced and are being successfully used to prevent infections and/or reduce disease severity, antiviral drugs are still needed to treat symptomatic patients [13]. Nevertheless, despite the fact that a few antivirals have already proved effective, the discovery of new drugs could have several advantages, e.g., greater effectiveness, broader range of action (also toward new variants), and lower cost, as well as for a better worldwide supply (for example, to the poorest countries) [14]. Despite the numerous articles about COVID-19, limited research has been carried out considering algae-derived bioactive metabolites as potential therapeutic agents. In this regard, this review tries to fill that gap by focusing on this aspect and how algal metabolites could inhibit SARS-CoV-2.

## 2. Structure of SARS-CoV-2 and Its Pathogenesis

**Figure 1:**
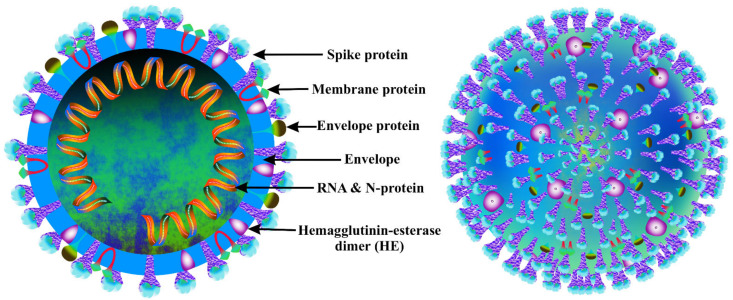
Cross-section (**left**) and outer structure (**right**) of the coronavirus.

Coronaviruses (CoVs) are single-stranded RNA viruses that can infect both humans and animals, causing a variety of respiratory and intestinal illnesses as well as life-threatening conditions, such as bronchiolitis and pneumonia [15]. CoV infections are especially dangerous for people who have a weakened immune system [16]. The International Committee on Virus Taxonomy (ICTV) classified the 2019 new coronavirus as SARS-CoV-2 [17,18]. Similar to the other coronaviruses, SARS-CoV-2 has a spherical shape and is made up of a capsid formed by the nucleocapsid protein (*N*-protein) with the viral DNA crammed inside. A cover is also present on the capsid, from which several structural proteins are produced. Membrane (*M*) proteins, spike (*S*) proteins, and envelope (*E*) proteins are three important structural components of the envelope shell [19]. The *S*-proteins protrude from the surface and mediate the virus’s entry into the host cell, stretching the virus’s crown-like appearance (Figure 1).

### 2.1. Interaction between the Host and the Coronavirus: The Heart of the Disease

**Figure 2:**
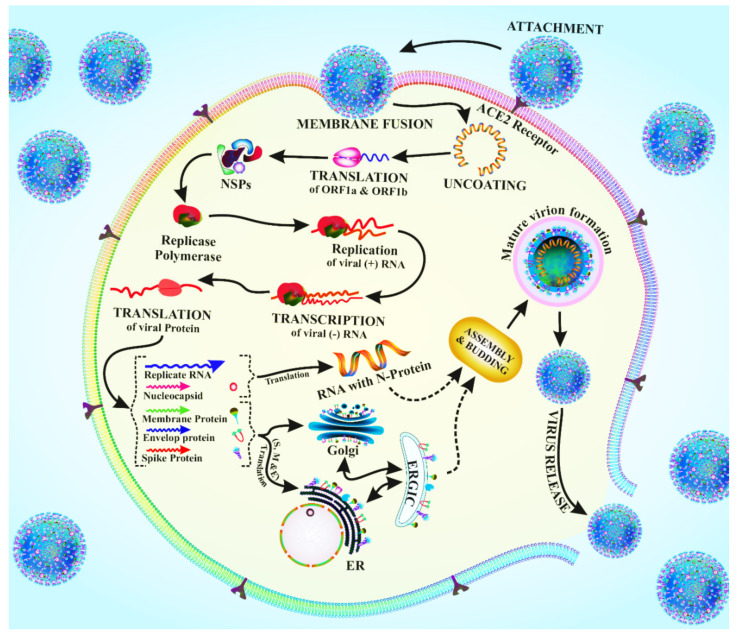
Schematic drawing of the viral entry and replication of the coronavirus.

The downstream section of the ORF1 (open reading frame 1) of coronaviruses contains unique genes that encode for structural proteins required for viral growth [19]. The spikes in the glycoprotein of the coronavirus are required for the viral attachment and penetration into host cells [19]. Cellular proteins, such as HAT (human airway trypsin-like proteases), cathepsins, and TMPRS2 (transmembrane protease serine 2), assist spike protein breaking, which leads to a deeper penetration of the coronavirus [16]. However, the coronavirus requires the crucial ACE2 (angiotensin-converting enzyme 2) receptor, which is also found in human cells, for viral entrance [20]. The spike protein interacts with the ACE2 receptor, inducing conformational changes that enhance the endosomal membrane fusion and the release of viral hereditary elements into the cytoplasm of the host cell [21]. The replication of CoV begins with the translation of ORF1a and ORF1b into pp1a and pp1ab polyproteins. Non-structural proteins (NSPs) are formed when these proteins are cleaved by proteases. The RTC (replicase–polymerase replication –transcription complex) is made up of NSPs that are involved in gRNA (viral genomic RNA) replication and subgenomic RNA transcription [21]. As a result of the continued production of structural proteins and other accessory proteins, accumulation of gRNA and viral proteins leads to the creation of fast-track virions [22]. The nucleocapsid is budded and then transferred by secretory vesicles, finally abandoning the host cell after the assembly process is completed. Budding is caused by the endoplasmic reticulum to Golgi intermediate complex (ERGIC) assembly process [22]. Understanding the mechanism of entry of the virus and its replication, as also schematized in Figure 2, shed light on the pathogenesis of the new coronavirus infection.

### 2.2. Pathogenesis of SARS-CoV-2

The pathogenesis of the new coronavirus infection is very similar to that of the SARS-CoV infection, which causes severe inflammation. SARS-CoV-2 is transmitted mostly by respiratory droplets, similarly to other coronaviruses that also cause respiratory illness [23]. Symptoms of COVID-19 infection include chills, dry cough, fever, sore throat, tiredness, and breathing difficulties. Cases of COVID-19 with severe ARDS (acute respiratory distress syndrome), which can lead to lung failure, are characterized by shortness of breath and low blood oxygen levels. Alveolar impairment, hyaline membrane formation, and modest microvesicular steatosis were seen in biopsy samples from liver, lung, and heart tissue of COVID-19 patients, indicating ARDS [24].

SARS-CoV-2 infects cells by initially forming a bond with the ACE2 protein and then entering them [25]. The host cell goes into pyroptosis as a result of the virus’s replication and release. PAMPs (pathogen-associated molecular patterns) and DAMPs (damage-associated molecular patterns) are released during pyroptosis and are recognized by surrounding cells, triggering the production of pro-inflammatory markers [26]. As a result, these protein molecules attract immune cells to the infection site, causing inflammation. Immune cells include monocytes, T cells, and macrophages [27]. The cells may disrupt the air–blood barrier by removing vascular endothelial cells and airway epithelial cells, resulting in collateral tissue damage. The coronavirus uses the high expression of the ACE2 receptor in endothelial cells and airway epithelial cells to penetrate inside the cell [28]. As a result, acute sickness is caused by both the viral infection and the overactive immunological response.

## 3. Diversity of Bioactive Metabolites and Their Potential Health Benefits as Dietary Supplements

Diet plays a critical role in the prevention of metabolic syndromes such as diabetes, cancer, and chronic diseases related with ageing [29,30]. Marine algae-derived bioactive metabolites, such as amino acids, peptides, fatty acids, lipids, carbohydrates, polysaccharides, sterols, polyphenols, photosynthetic pigments, carrageenan, agar, fucoidan, laminaran, naviculan, vitamins, and minerals, exert a persuasive antioxidant activity that has a beneficial effect in fighting the aforementioned diseases [5]. In addition to preventing these chronic diseases, some of these metabolites have been also tested as antiviral agents against several virus-associated infections [8]. Moreover, both marine microalgae and macroalgae (seaweeds) contain high amounts of amino acids and vitamins [31,32,33,34], which might help to fight viral diseases [35].

### 3.1. Cyanobacteria- and Algae-Derived Bioactive Metabolites and Their Potential Role as Antiviral Agents

**Figure 3:**
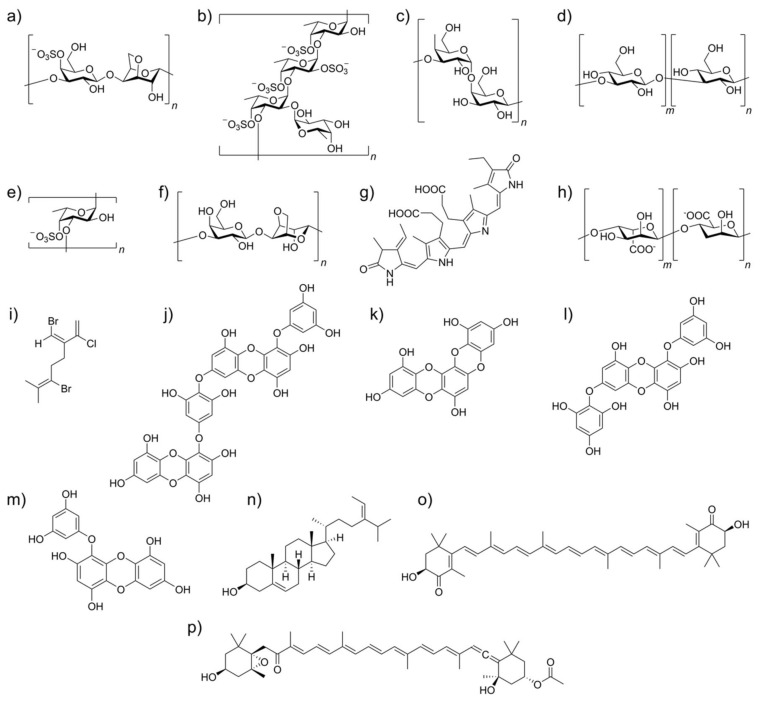
Molecular structures of algae-derived bioactive metabolites which can modulate viral inhibition and act as immunomodulators: (**a**) κ-carrageenan; (**b**) a fucoidan from *Laminaria saccharina*; (**c**) galactan; (**d**) laminaran; (**e**) a sulfated fucan; (**f**) agar; (**g**) C-phycocyanin; (**h**) alginate; (**i**) a halogenated monoterpene; (**j**) diecol; (**k**) dioxinodehydroeckol; (**l**) 7-phloroeckol; (**m**) eckol; (**n**) fucosterol; (**o**) astaxanthin; (**p**) fucoxanthin.

The molecular structure of the main algae-derived bioactive metabolites and their potential antiviral activities against human pathogenic viruses and their mode of action are given in Figure 3 and Table 1, respectively.

Marine algae are rich in polysaccharides that exhibit antiviral activities [6,12]. Polysaccharides extracted from *Spirulina platensis* have shown antiviral activity against various viruses, such as measles virus, HSV type 1, influenza A virus, HCMV, mumps virus, and HIV-1, in several human cell lines [12]. Calcium spirulan from this cyanobacterium repressed viral replication by inhibiting viral entry into the host cell. The chief constituents of calcium spirulan are calcium and sulfated glucuronic acid, xylose, glucose, mannose, galacturonic acid, ribose, fructose, rhamnose, and galactose, which displayed antiviral effects due to the molecular conformation adopted by chelating the calcium ions with the sulfate groups [12]. Serum samples from animals given calcium spirulan also showed long-term antiviral activity against HSV-1 and HIV-1 [62].

Moreover, marine algae are also rich in sulfated polysaccharides that prevent the replication of viruses, and they have been clinically tested, e.g., against HSV-1 [63,64]. Sulfated polysaccharides inhibit antiviral pathways and act as potential replication inhibitors of retroviruses such as HIV-V [36]. Carrageenan is a common polysaccharide with recognized activity against viral infections. Carrageenan is a sulfated polymer isolated from red algae, such as *Gigartina*, *Chondrus*, *Eucheuma*, and *Hypnea*, able to block viral entry by inhibiting binding to the host cells [37]. It was also shown to limit the reproduction of DENV in mosquitoes and in mammalian cells [36]. Moreover, it possesses an operative role against HPV (human papillomavirus), which leads to genital warts and cervical cancer [65]. Carrageenans with low molecular weight (3, 5, and 10 kDa) show a repressing effect against the influenza virus [38,39]. In addition, the administration of a carrageenan-based nasal spray (Iota-carrageenan), also recognized as “super-shedders”, is effective against the common cold, by improving viral clearance and reducing cold duration. A carrageenan extracted from a red alga (*Schizymenia pacifica*) was able to hinder the functions of reverse transcriptase in avian and mammalian retroviruses. Furthermore, it also prevented the binding between the host and the virus in the early stages of the infection [40]. Extracellular sulfated polysaccharides isolated from *Cochlodinium polykrikoides* reduced blood coagulation and inhibited influenza A and B virus in MDCK cells, respiratory virus of types A and B in Hep-2 cells, and immunodeficiency virus of type-1 in MT-4 cells [53]. p-KG03, another sulfated exo-polysaccharide derived from *Gyrodinium impudicum*, showed antiviral properties against EMCV (encephalomyocarditis virus) without toxicity in HeLa cells [54]. In addition, it inhibited duplication of influenza A virus by targeting adsorption and integration into the host cell [54].

Alginates hinder HIV replication by reducing DNA polymerase activity and inhibiting viral reproduction by downregulating the functioning of reverse transcriptase, disrupting the viral adsorption, and educating host cell defense mechanisms [8]. Sulfated polymannuroguluronate (SPMG) inhibits HIV-1 infection by preventing viral glycoprotein gp120 from attaching to CD4 molecules on the surface of T cells [8]. In addition, it prevents viral multiplication and syncytium formation between infected and healthy cells. The alginate-derived marine polysaccharide drug 911 reduced HIV-1 infection in MT4 cells and chronic infection in H9 cells [66,67].

Extracellular polysaccharides, such as galactosides isolated from the red alga *Agardhiella tenera*, exhibited antiviral properties against DENV, HSV-1, HSV-2, HIV-1, HIV-2, and Hep A virus (hepatitis A virus) [41]. Galactans isolated from *Callophyllis variegate* showed antiviral activity against HSV-1, HSV-2, and DENV-2 with low cytotoxicity [42]. The antiviral efficacy of a sulfated galactan isolated from *Schizymenia binderi* against HSV-1 and HSV-2 was also demonstrated [43].

Fucans are high-molecular-weight polysaccharides classified into three subgroups as fucoidans, glycuronogalactofucans, and xylofucoglycuronans [44]. Sulfated fucans isolated from *Dictyota mertensii, Lobophorava riegata, Fucus vesiculosus,* and *Spatoglossum schroederi* prevented HIV infection by slowing the action of reverse transcriptase. [44]. In the BHK-21 cell line of baby hamster kidney cells, a fucan polysaccharide produced from *Cladosiphon okamuranus* inhibited DENV-2 infection [45]. Compared to known synthetic drugs, the fucose polysaccharides compound (MC26) isolated from *Sargassum piluliferum* showed promising anti-influenza viral activity with extremely low cytotoxicity both in vivo and in vitro [46]. Fucoidans isolated from brown algae, such as *Undaria pinnatifida*, *Adenocytis utricularis*, *Cystoseira indica*, and *Stoechospermum marginatum*, showed antiviral activity in vivo and in vitro against both DNA and RNA viruses, such as HSV-1 and HSV-2, cytomegalovirus, and DENV, by inhibiting viral interaction with the cells and the syncytium formation. [47]. Moreover, fucoidans extracted from *Turbinaria decurrens* and *Dictyota bartayesiana* displayed potential inhibitory efficacy against HIV [47,48]. Laminarin from *Laminaria japonica, Eisenia bicyclis,* and *Ecklonia kurome* yields two derivatives, i.e., that with glucose residues and that with a terminal D-mannitol residue, which also prevented the adsorption of HIV reverse transcriptase [49,50].

HSV-1, HSV-2, influenza A virus, and HCMV all have carbohydrates as cellular receptors, and Nostoflan, an acidic polysaccharide produced from *Nostoc flagelliforme*, showed antiviral properties against those viruses, also preventing the initial stage of the virus infection [51]. Naviculan is a type of sulfated polysaccharide derived from *Navicula directa* which is composed of xylose, galactose, fucose, rhamnose, sulfate, and mannose, and it displayed novel antiviral properties against HSV-1, HSV-2, and influenza A virus. Moreover, it hindered the combination of gp160 HIV-expressing cells and the CD4 receptor [52]. 

Phycobiliproteins extracted from *Spirulina* are well recognized for their anti-inflammatory, antioxidant, and antimicrobial activities, and they also exhibit antiviral properties [55,56]. Oral administration (5, 12.5, and 25 mg/kg every 4 h) of a *Spirulina* extract reduced viral infection in female BALB/c mice previously inoculated with H1N1 virus. Moreover, it increased the survival rate by 20%, 40%, and 60%, respectively [55]. C-phycocyanin and allophycocyanin made up 50% and 10%, respectively, of the total protein fraction and contributed to the antiviral properties of the *Spirulina* extract [57]. Extracts of Brazilian raw marine algae also exhibited an extraordinary anti-therapeutic action. In addition, they also inhibited the viral activity of HSV-1 (86.1%) with greater efficacy than HSV-2 (55.5%) [58]. An aqueous extract from *Laurencia obtusa* red algae showed in vitro antiviral activity by hindering the reproduction of influenza B, A (H3N2), and A (H1N1) viruses [56]. Furthermore, extracts of Egyptian *Ulva lactuca* and *Cystoseira myrica* seaweeds displayed notable antiviral activities against Coxsackie B4, hepatitis A, HSV-1, and HSV-2 [59]. Cold water extract from *Arthrospira platensis* exhibited an antiviral effect against mice infected with H1N1 (influenza A/WSN/33) virus [55,56]. Allophycocyanin isolated from *S. platensis* neutralized the enterovirus 71-induced cytopathic effect in human rhabdomyosarcoma cells by delaying the synthesis of viral RNA and diminishing the apoptotic process along with DNA fragmentation, reduction in membrane damage, and sub-G1 phase cell decline [57]. It also delayed Pheophorbide-like compounds isolated from *Dunaliella primolecta* that prevent the cytopathic effect of HSV-1 virus during adsorption, entry, and invasion into host cells [60]. Phlorotannins from *Ecklonia cava* inhibited syncytia formation, lytic cycle effects, and viral p24 antigen production both in vitro and in vivo. They also effectively inhibited the HIV-1 reverse transcriptase enzyme [61].

### 3.2. Algae-Derived Lectins: A Promising Source of Antiviral Activity

Lectins are highly specific carbohydrate-binding proteins responsible for many recognition processes at the cellular and molecular levels, as well as for the binding of fungi and viruses to the host cell. Algae are a rich source of lectins, which are used in biomedical research for their antiviral, antinociceptive, anti-inflammatory, and anti-tumor activity, among others [68,69].

Algal lectins bind reversibly to receptors of viruses in a non-covalent and very specific way. Cyanovirin, a special class of algal lectin (molecular weight of 11 kDa) extracted from *Nostoc ellipsosporum*, binds to the glycoprotein (gp120) envelope and inhibits the entry of several viruses, such as HIV-1, HIV-2, SIV (simian immunodeficiency virus), and feline immunodeficiency virus [70]. Cyanovirin works after the attachment between virus and host cell is complete or in the entry course after the CD-4 binding step. In CD4 T cells, microvirin isolated from *Microcystis aeruginosa* reduces initiation markers such as CD69, CD25, and HLA-DR. It also prevents HIV-1-infected T cells from forming syncytium with healthy CD4 T cells [71]. Griffithsin, a type of algal lectin identified from *Griffithsia* sp., is thought to be the most potent HIV inhibitor, with an IC_50_ value in the picomolar range [72]. Additionally, it inhibits HCV (hepatitis C virus) infection of Huh-7 hepatoma cells in vitro and HCV infection of mice with human primary hepatocytes in the liver in vivo [73]. It also binds to the HCV envelope glycoproteins (E1 and E2), preventing the virus from infecting human hepatocytes [73,74]. Furthermore, griffithsin protects mice infected with genital HSV-2 and prevents cell-to-cell transmission without causing harm [75]. It is also known to prevent SARS-CoV infection by specifically binding to the *S*-protein [76] and MERS-CoV infection in vitro at the level of viral entry [77]. Scytovirin isolated from *Scytonema varium* displayed antiviral activities against Zaire ebolavirus, Marburg virus, HIV, and SARS-CoV [78]. Mice infected with the Ebola virus received hypodermic scytovirin (30 mg/kg/day) every 6 h, resulting in a 90% survival rate [79]. Scytovirin was shown to bind with high affinity to oligosaccharides rich in mannose on the glycoprotein envelope, thus blocking viral entry into the target cell [80]. The red alga *Kappaphycus alvarezii* and the green alga *Boodlea coacta* have higher levels of mannose-specific lectin (i.e., KAA-2) and agglutinin (i.e., BCA), respectively. Multiple strains of influenza virus, including the pandemic H1N1-2009, are inhibited by these lectins. Furthermore, they also restrict the entry of the virus into the host cell after the direct binding of hemagglutinin (HA) to the envelope of the virus [60,81,82]. Marine algae-derived lectins with promising antiviral properties and their mode of action are summarized in Table 2.

### 3.3. Algae-Derived Bioactive Metabolites and Their Antiviral Potential against SARS-CoV-2

The development of antiviral drugs has paid much attention to controlling the host defense and targeting the viral infection process [81]. Blocking the signaling transduction pathways of human cells helps to control viral replication. The antiviral drugs targeting SARS-CoV-2 have been mainly synthesized with the primary goal of inhibiting RNA synthesis and replication by hindering the viral binding to human cell receptors [82]. With drug toxicities as a major concern, screening and validation of natural bioactive metabolites may enlighten the development of new pharmaceutical drugs as antiviral mediators against SARS-CoV-2.

Algae-derived bioactive metabolites such as lectins and polysaccharides, including carrageenan, nostoflan, microvirin, galactans, and cyanovirin, have been proposed as potential drugs against SARS-CoV-Sulfated polysaccharides bind to SARS-CoV-2 *S*-protein and function as diversions, preventing *S*-protein binding to the heparin sulfate co-receptor in host tissues and thereby preventing viral infection [83]. Polysaccharides from seaweed can hinder the viral life cycle by deactivating virions before viral infection [84]. Carrageenan and chitosan are types of polysaccharides that have shown virucidal actions by inhibiting viral infection [83]. These polysaccharides reduce viral infection by blocking several mechanisms, such as viral absorption, virus uncoating, incorporation, transcription, and replication. Nagle et al., reported that exopolysaccharides with carrageenan and sulfated polysaccharides isolated from *Porphyridium* prevent virus binding on the host cell. Modified chitosan significantly prevents against human coronaviruses HCoV-OC43, HCoV-229E, HCoV-NL63, and HCoVHKU1 [8,85]. Chitosan also inhibits low-pathogenic human coronaviruses [86]. Moreover, fucoidan and sulfated rhamnan downregulate the expression and stimulation of the epidermal growth factor receptor pathway to fight coronavirus [55]. Halitunal is a novel diterpene aldehyde isolated from *Halimeda tuna* which displayed in vitro antiviral activities against the murine coronavirus A59 [87]. Griffithsin extracted from red algae displayed antiviral properties by binding the oligosaccharides on the surface of MERS-CoV and SARS-CoV viral spike glycoproteins [76]. In addition, griffithsin also prevented the in vitro action of CoVs, such as HCoV-229E, HCoV-OC43, and HCoV-NL63, and in vivo against SARS-CoV-infected mice. It also performed very effectively against SARS-CoV-2 by inhibiting viral entry, integrase activity, reverse transcriptase activity, and protease activity. Ulvansare, a polysaccharide extracted from a green alga, is considered a potential therapeutic mediator against SARS-CoV-2 [85]. Phycocyanobilins, phycoerythrobilins, and folic acid isolated from *Arthrospira* displayed potential antiviral efficacy against SARS-CoV-2 [88]. In silico studies revealed that the bioactive compounds isolated from *Gracilaria corticata*, *Laurencia papillosa*, and *Grateloupia filicina* could possess therapeutic efficacy against SARS-CoVs, including SARS-CoV-2 [88,89].

### 3.4. Drug Synergy: The Emerging Therapy against SARS-CoV-2

Drug synergy has provided novel ideas for improving the efficacy of the preliminary anti-SARS-CoV-2 drugs [90]. A combination of polysaccharides and other bioactive metabolites of algal origin could be used as potent antiviral mediators against SARS-CoV-2 [55]. Astaxanthin derived from the microalga *Haematococcus pluvialis* has been shown to possess anti-inflammatory, anti-oxidative, immune booster, and immunomodulator capacities against respiratory disorder models and may have beneficial effects in combination with primary antiviral drugs on COVID-19 patients [91]. Algal lectins, ulvans, carrageenan, and fucoidans isolated from red, green, and brown algae also possess many health-promoting abilities and could be tested as synergistic therapeutic agents to prevent and treat COVID-19 [91]. Dieckol aphlorotannins isolated from *Ecklonia cava* showed the most potent 3CLpro *trans*-/*cis*-cleavage inhibitory action against SARS-CoV in a dose-dependent manner without toxicity [92].

### 3.5. Algae-Derived Lectins as Therapeutics against SARS-CoV-2

Glycoproteins are oligosaccharide chains attached to proteins, and they have numerous biological activities and also antiviral properties [76]. Currently, glycotherapy represents an interesting approach in viral research, because the initial attachment between the virus and the host cell occurs through the viral spike proteins and the glycoproteins on the cell surface. Glycans are attached to the spike protein of SARS-CoV-2, thus masking it. The *S*-protein of the SARS-CoV-2 holds 66 glycosylation sites, and hACE2 (human angiotensin-converting enzyme 2) is engaged as receptor for the entry of SARS-CoV-2 [76]. Lectins from red algae are rich in mannosylated *N*-glycans, and they can inhibit coronavirus infectivity [68]. Red algae-derived high-mannose binding lectin griffithsin is expected to inhibit SARS-CoV entry via spike glycoprotein binding [93]. In addition, griffithsin did not present any toxicity to the host cell, even at high concentration [93].

### 3.6. Cyanobacterial and Algal Metabolites: The Gift of Future Nutraceuticals?

*Spirulina*, a cyanobacterium, is rich in bioactive metabolites, such as essential fatty acids, phenolic acids, sulfated polysaccharides, and vitamin B12 [94]. These products are manufactured and commercially available as dietary supplements [95]. Nutraceuticals and bioactive metabolites from *Spirulina* are well known for their antioxidant, antiviral, anti-inflammatory, and immunomodulatory activities [95]. Natural ACE-inhibiting antioxidants, such as ACE inhibitory peptides, and antiviral agents, such as calcium spirulan and phycocyanobilin nutraceuticals, may also be included in clinical trials. These compounds can strengthen the immune system, helping to prevent the disease and to treat post-COVID-associated complications [12]. The role of algae-derived nutraceuticals in SARS-CoV-2 infections is still limited and requires much attention for further development and use. Sulfated polysaccharides isolated from the red algae *Porphyridium* sp. act as nutraceuticals and exhibit potent antiviral capacity [84]. They could be also exploited as wrapping films on clean items for preventing COVID-Nevertheless, clinical research on animal models and human trials is still needed to further understand the potential exploitation of algal bioactive metabolites as COVID-19 nutraceuticals [94,96,97].

## 4. Immunomodulatory Activity of Bioactive Metabolites against SARS-CoV-2 by Microbiota-Based Therapy

**Figure 4:**
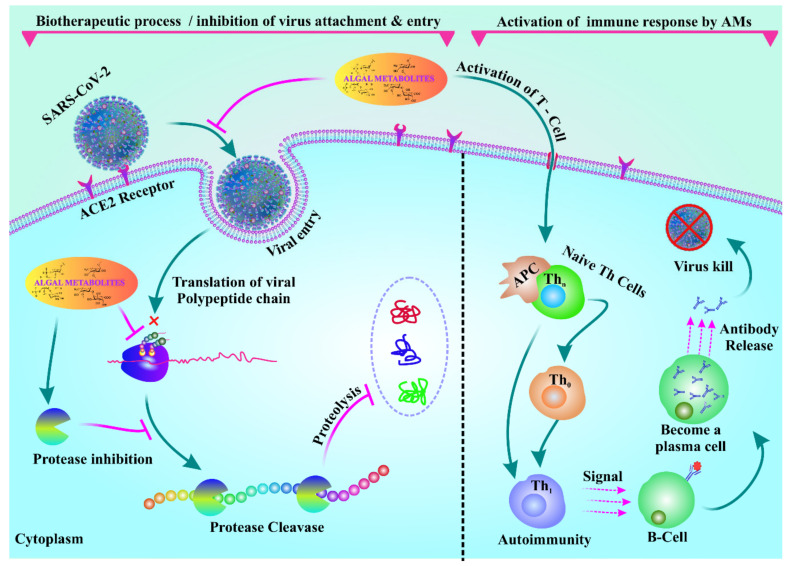
Algal metabolites inhibit adhesion and entry of the virus. Algal metabolites can also activate immune responses in COVID-19 patients by activating T cells.

Immunity is a major concern in people suffering from COVID-After drug treatment, patients gradually become immunocompromised [98]. SARS-CoV-2 causes gastrointestinal disorder in nearly 20% of patients who suffer from it [99]. Even more striking, Effenberger et al., reported that 61% of patients suffer from gastrointestinal upset, diarrhea, and nausea. Therefore, natural immunomodulators from algae seem to be promising in this context. A recent pilot study on the composition of the microbiome in stool samples from 15 hospitalized COVID-19 patients revealed poor gut health compared to that of healthy individuals [100]. On the other hand, a vigorous gut microbiome is essential to modulate antiviral immunity, but this can only be achieved by improving gut flora [100]. In such circumstances, algae-based bioactive metabolites could be used as food supplements capable of improving the gut microbiota, thus potentially reducing the incidence of SARS-CoV-ACE2-associated gut microbiota symbiosis plays a key role in improving antiviral immunity by stimulating interferon production, decreasing immunopathology, and increasing natural killer (NK) cytotoxicity in COVID-19 patients [101]. Marine bioactive metabolites, such as carrageenans, fucoidans, alginates, polyphenols, luminaries, carotenoids, phlorotannins, and fatty acids, can trigger the human gut microbiota and maintain host health by controlling proper metabolism, epithelial barrier integrity, and immune system efficacy when used as prebiotics and nutritional food supplements. Seaweed is rich in vitamins and minerals that can be used as dietary supplements for COVID-19 patients. Furthermore, carotenoids, phytosterols, vitamins, and fatty acids isolated from different microalgae species showed promising immunomodulation activity [5]. Consumption of *Chlamydomonas reinhardtii* modulates human gastrointestinal disorders, such as diarrhea, gas, and bloating, and also the microbiota composition [102]. *Spirulina* controls the immune systems by modulating the gut microbiota and upregulates toll-like receptors 2 and 4 (TLR2 and TLR4) [103]. Pectin isolated from *Spirulina* also modulates the gut microbiota and triggers immunity [104]. β-Glucan isolated from algae relieves gut health in weaned pigs with *E. coli* infection [105]. Sulfated polysaccharides isolated from *Ascophyllum nodosum* stimulate an abundance of beneficial firmicutes and bacteroidetes [106]. Algae-based polysaccharides, such as carrageenan, alginate, and agarose, can also provide a beneficial effect on the human gut microbiota and also on gut health in general [85]. *Sargassum muticum* and *Osmundea apinnatifida* extracts exert a beneficial effect on the human gut microbiota, and they have been used as novel functional foods [107]. The immunomodulatory effect of phycobiliproteins isolated from *Spirulina* is also promising [95]. Viral immune responses against COVID-19 and targets of common dermatologic immunomodulators are shown in Figure 4.

## 5. Conclusions and Future Perspectives

Infectious diseases caused by RNA viruses have been a serious threat to global health for years. Currently, the SARS-CoV-2 virus menaces the entire world with the COVID-19 pandemic. Despite vaccination, novel therapeutic strategies are still needed to maintain human health. Algae contain several bioactive metabolites, such as sulfated polysaccharides, polyphenols, and lectins, which possess robust antiviral activity and immunostimulating effects. In this regard, the bioactive metabolites of marine algae could represent a promising alternative to fight COVID-In addition, marine algae are rich in antioxidant molecules that could help to reduce the oxidative stress associated with the infection caused by SARS-CoV-2 [108]. These bioactive metabolites have been also evaluated and exploited as microbiota-based therapeutic agents and immunomodulators, which can also enhance treatment against SARS-CoV-2. 

With these premises, algal metabolites could be an ideal candidate for preliminary treatment, in conjunction with other pharmacological therapies, of COVID-19. 

Although the seaweed aquaculture and the extraction process should be optimized, the availability of algal biomolecules represents a cost-effective and ecologically benign approach. Furthermore, exploitation of nanoformulations could significantly lower the amount of active principal ingredient required for treatment.

Eventually, identification of bioactive molecules could also be exploited for creating, together with an in silico approach or by using high-throughput technology, novel synthetic prototypes to be tested in vitro and in vivo.

Nevertheless, with the limited evidence available, more in vivo and clinical studies are essential to establish the most effective natural algal bioactive metabolites and immunomodulators against SARS-CoV-2.

## Figures and Tables

**Table 1:** Cyanobacterial and algal-derived bioactive metabolites and their potential antiviral activities against human pathogenic viruses and their mode of action.

Bioactive Compounds	Cyanobacterial/ Algal Sources	Viruses Involved	Mode of Action	References
Polysaccharides	*Spirulina platensis*	Measles virus, HSV-1, HCMV, influenza A, mumps virus, HIV-1	Blockage of viral replication by inhibiting the penetration of the virus into the host cell	[12]
Calcium-spirulan				
Sulfated polysaccharides	*Brown algae*	HSV-1, HIV	Prevention of the viral replication	[36]
	*Chlorella vulgaris, Cochlodinium polykrikoides*, *Porphyridium* sp.	Influenza A and B, RSV-A, RSV-B, parainfluenza-2	Inhibition of the cytopathic effect; inhibition of PMN migration toward chemoattractant molecules; partial blocking of the adhesion to endothelial cells	
Carrageenan	*Chondrus, Gigartina, Hypnea, Eucheuma*	Dengue virus (DENV), HPV	Blockage of the viral entry by inhibiting their binding to the host cell	[37]
Low molecular weight of carrageenans	*Chondrus, Gigartina, Hypnea, Eucheuma*	Influenza virus	Inhibitory effect	[38,39]
Carrageenan	*Schizymenia pacifica*	Avian retrovirus (avian myeloblastosis virus), mammalian retrovirus (rauscher murine leukemia virus)	Hindering of the function and replication of reverse transcriptase and prevention of the viral binding to the host cell at the initial stages of infection	[40]
	*Gigartina skottsbergii*	Influenza virus, DENV, HSV-1, HSV-2, HPV, HRV, HIV	Inhibition of the binding or the internalization of viruses into host cells (Stage I, II, III)	
Alginates	*Laminaria hyperborea, Laminaria digitata, Laminaria japonica*	HIV, IAV, HBV	Inhibition of the viral HIV reproduction by downregulating the activity of reverse transcriptase	[8]
Sulfated polymannuroguluronate		HIV-1	Shielding of the viral glycoprotein and blockage of the viral duplication	
Galactose	*Agardhiella tenera*	HSV-1, HSV-2, DENV, HIV-1, HIV-2, Hep A	Antiviral properties	[41]
Galactans	*Callophyllis variegate*	HSV-1, HSV-2, DENV-2	Antiviral activity with low cytotoxicity	[42]
	*Callophyllis variegata, Agardhiella tenera, Schizymenia binderi, Cryptonemia crenulata*	HSV-1, HSV-2, HIV-1, HIV-2, DENV, HAV	Blockage of virus adhesion and replication into host cells	
Sulfated galactan	*Schizymenia binderi*	HSV-1, HSV-2	Antiviral activity with low cytotoxicity	[43]
Fucan	*Adenocytis utricularis, Undaria pinnatifida, Stoechospermum marginatum, Cystoseira indica, Cladosiphon okamuranus, Fucus vesiculosus*	HSV-1, HSV-2, HCMV, VSV, Sindbis virus, HIV-1	Inhibition of cell adhesion (Stage I), blockage of reverse transcriptase	[44]
Sulfated fucans	*Dictyota mertensii, Lobophora variegata, Fucus vesiculosus, and Spatoglossum schroederi*	HIV	Antiviral activities by blocking the activity of reverse transcriptase	
Fucan polysaccharide	*Cladosiphon okamuranus*	DENV-2	Inhibition of the infection	[45]
Fucose polysaccharides (MC26)	*Sargassum piluliferum*	Influenza virus	Antiviral activity with low cytotoxicity	[46]
Fucoidans	*Adenocytis utricularis, Undaria pinnatifida, Stoechospermum marginatum, Cystoseira indica*	HSV-1, HSV-2, DENV, cytomegalovirus	Blockage of the viral interaction with the cell and inhibition of syncytium formation	[47]
	*Dictyota bartayesiana and Turbinaria decurrens*	HIV	Antiviral activity	[47,48]
Laminarin	*Laminaria japonica, Ecklonia kurome, Eisenia bicyclis*	HIV	Prevention of the adsorption of HIV reverse transcriptase	[49,50]
	*Fucus vesiculosus, Saccharina longicruris, Ascophyllum nodosum*		Blockage of reverse transcriptase	
Nostoflan	*Nostoc flagelliforme*	HSV-1, HSV-2, HCMV, influenza A	Antiviral activity at the initial stage of viral infection	[51]
Naviculan	*Navicula directa*	HSV-1, HSV-2, influenza A	Antiviral activity	[52]
A1 and A2 polysaccharide	*Cochlodinium polykrikoides*	Influenza A and B	Antiviral activity	[53]
p-KG03	*Gyrodinium impudicum*	EMCV	Antiviral activity	[54]
		Influenza A	Inhibition of viral duplication by targeting adsorption and incorporation into the host cell	
Phycobiliproteins	*Arthrospira platensis*	Influenza A/WSN/33 (H1N1) virus	Inhibition of the viral infection	[55,56]
C-phycocyanin and allophycocyanin	*Spirulina*		Antiviral activities	[57]
Crude extracts	Brazilian marine algae	HSV-1, HSV-2	Antiviral activity	[58]
Red algal aqueous extract	*Laurencia obtuse*	Influenza B, A (H3N2), and A (H1N1)	In vitro antiviral activity by hindering the reproduction	[56]
Crude extracts	*Ulva lactuca and Cystoseira myrica*	Coxsackie B4, hepatitis A, HSV-1, HSV-2	Antiviral activity	[59]
Allophycocyanin	*S. platensis*	Influenza B	Blockage of the viral entry	[57]
Pheophorbide	*Dunaliella primolecta*	HSV-1	Inhibition of the viral adsorption and invasion	[60]
Phlorotannins	*Ecklonia cava*	HIV-1	Prevention of syncytia formation, lytic effects and viral p24 antigen production in vitro and in vivo	[61]

**Table 2:** Marine algae-derived lectins with promising antiviral properties and their mode of action.

Algal Lectins	Algal Sources	Viruses Involved	Mode of Action	References
Cyanovirin	*Nostoc ellipsosporum*	HIV-1, HIV-2, SIV, feline immunodeficiency virus	Inhibition of the viral entry by binding to the gp120	[70]
Microvirin	*Microcystis aeruginosa*	CD4 T HIV-1	Reduction of initiation markers such as CD69, CD25, and HLA-DR by syncytium formation with healthy CD4 T cells	[71]
Griffithsin	*Griffithsia* sp.	HIV	Potent antiviral activity both in vivo and in vitro	[73]
		HCV (hepatitis C Virus) in Huh-7 hepatoma cell		
		Hepatitis C Virus	Prevention of the infection in human hepatocytes	[73,74]
		SARS-CoV	Prevention of the infection by binding to the *S*-protein	[76]
Scytovirin	*Scytonema varium*	Zaire ebolavirus, Marburg virus, HIV, and SARS-CoV	Binding to the viral coat proteins gp120, gp160, and gp41 but not to cellular receptor CD4 or other tested proteins	[78]
		Ebola virus		
Mannose-specific lectin, agglutinin, and KAA-2, BCA	*Kappaphycus alvarezii, Kappaphycus alvarezii, Boodlea coacta*	Influenza virus	Inhibition of viral entry	[60,81,82]

## References

[1] Guo Y.R., Cao Q.D., Hong Z.S., Tan Y.Y., Chen S.D., Jin H.J., Tan K.S., Wang D.Y., Yan Y. (2020). The origin, transmission and clinical therapies on coronavirus disease 2019 (COVID-19) outbreak—An update on the status. Mil. Med. Res..

[2] Elengoe A. (2020). COVID-19 Outbreak in Malaysia. Osong Public Health Res. Perspect..

[3] Singhal T. (2020). A Review of Coronavirus Disease-2019 (COVID-19). Indian J. Pediatr..

[4] Pradhan B., Maharana S., Bhakta S., Jena M. (2021). Marine phytoplankton diversity of Odisha coast, India with special reference to new record of diatoms and dinoflagellates. Vegetos.

[5] Pradhan B., Nayak R., Patra S., Jit B.P., Ragusa A., Jena M. (2021). Bioactive Metabolites from Marine Algae as Potent Pharmacophores against Oxidative Stress-Associated Human Diseases: A Comprehensive Review. Molecules.

[6] Pradhan B., Patra S., Nayak R., Behera C., Dash S.R., Nayak S., Sahu B.B., Bhutia S.K., Jena M. (2020). Multifunctional role of fucoidan, sulfated polysaccharides in human health and disease: A journey under the sea in pursuit of potent therapeutic agents. Int. J. Biol. Macromol..

[7] Shi Q., Wang A., Lu Z., Qin C., Hu J., Yin J. (2017). Overview on the antiviral activities and mechanisms of marine polysaccharides from seaweeds. Carbohydr. Res..

[8] Wang W., Wang S.X., Guan H.S. (2012). The antiviral activities and mechanisms of marine polysaccharides: An overview. Mar. Drugs.

[9] Dhar D., Mohanty A. (2020). Gut microbiota and Covid-19- possible link and implications. Virus Res..

[10] Alam S., Sarker M.M.R., Afrin S., Richi F.T., Zhao C., Zhou J.R., Mohamed I.N. (2021). Traditional Herbal Medicines, Bioactive Metabolites, and Plant Products Against COVID-19: Update on Clinical Trials and Mechanism of Actions. Front. Pharmacol..

[11] Ratha S.K., Renuka N., Rawat I., Bux F. (2021). Prospective options of algae-derived nutraceuticals as supplements to combat COVID-19 and human coronavirus diseases. Nutrition.

[12] Hayashi K., Hayashi T., Kojima I. (1996). A natural sulfated polysaccharide, calcium spirulan, isolated from *Spirulina platensis*: In vitro and ex vivo evaluation of anti-herpes simplex virus and anti-human immunodeficiency virus activities. AIDS Res. Hum. Retroviruses.

[13] Tregoning J.S., Flight K.E., Higham S.L., Wang Z., Pierce B.F. (2021). Progress of the COVID-19 vaccine effort: Viruses, vaccines and variants versus efficacy, effectiveness and escape. Nat. Rev. Immunol..

[14] Menéndez J.C. (2022). Approaches to the Potential Therapy of COVID-19: A General Overview from the Medicinal Chemistry Perspective. Molecules.

[15] V’Kovski P., Kratzel A., Steiner S., Stalder H., Thiel V. (2021). Coronavirus biology and replication: Implications for SARS-CoV-*Nat*. Rev. Microbiol..

[16] Subbarao K., Mahanty S. (2020). Respiratory Virus Infections: Understanding COVID-19. Immunity.

[17] Gorbalenya A.E., Baker S.C., Baric R.S., de Groot R.J., Drosten C., Gulyaeva A.A., Haagmans B.L., Lauber C., Leontovich A.M., Neuman B.W. (2020). The species Severe acute respiratory syndrome-related coronavirus: Classifying 2019-nCoV and naming it SARS-CoV-2. Nat. Microbiol..

[18] Liu Y.C., Kuo R.L., Shih S.R. (2020). COVID-19: The first documented coronavirus pandemic in history. Biomed. J..

[19] Huang Y., Yang C., Xu X.F., Xu W., Liu S.W. (2020). Structural and functional properties of SARS-CoV-2 spike protein: Potential antivirus drug development for COVID-19. Acta Pharmacol. Sin.

[20] Parasher A. (2021). COVID-19: Current understanding of its Pathophysiology, Clinical presentation and Treatment. Postgrad. Med. J..

[21] Wan Y., Shang J., Sun S., Tai W., Chen J., Geng Q., He L., Chen Y., Wu J., Shi Z. (2020). Molecular Mechanism for Antibody-Dependent Enhancement of Coronavirus Entry. J. Virol..

[22] Chatterjee S.K., Saha S., Munoz M.N.M. (2020). Molecular Pathogenesis, Immunopathogenesis and Novel Therapeutic Strategy Against COVID-*Front*. Mol. Biosci..

[23] Jin Y., Yang H., Ji W., Wu W., Chen S. (2020). Virology, Epidemiology, Pathogenesis, and Control of COVID-19. Viruses.

[24] Huppert L.A., Matthay M.A., Ware L.B. (2019). Pathogenesis of Acute Respiratory Distress Syndrome. Semin. Respir. Crit. Care Med..

[25] Perrotta F., Matera M.G., Cazzola M., Bianco A. (2020). Severe respiratory SARS-CoV2 infection: Does ACE2 receptor matter?. Respir. Med..

[26] Tay M.Z., Poh C.M., Rénia L., MacAry P.A., Ng L.F.P. (2020). The trinity of COVID-19: Immunity, inflammation and intervention. Nat. Rev. Immunol..

[27] Schijns V., Lavelle E.C. (2020). Prevention and treatment of COVID-19 disease by controlled modulation of innate immunity. Eur. J. Immunol..

[28] Flerlage T., Boyd D.F., Meliopoulos V. (2021). Influenza virus and SARS-CoV-2: Pathogenesis and host responses in the respiratory tract. Nat. Rev. Microbiol..

[29] Kouvari M., D’Cunha N.M., Travica N., Sergi D., Zec M., Marx W., Naumovski N. (2022). Metabolic Syndrome, Cognitive Impairment and the Role of Diet: A Narrative Review. Nutrients.

[30] Alkhatib D.H., Jaleel A., Tariq M.N.M., Feehan J., Apostolopoulos V., Cheikh Ismail L., Stojanovska L., Dhaheri A.S.A. (2021). The Role of Bioactive Compounds from Dietary Spices in the Management of Metabolic Syndrome: An Overview. Nutrients.

[31] Pradhan B., Patra S., Dash S., Nayak R., Behera C., Jena M. (2021). Evaluation of the anti-bacterial activity of methanolic extract of *Chlorella vulgaris* Beyerinck [Beijerinck] with special reference to antioxidant modulation. Futur. J. Pharm. Sci..

[32] Pradhan B., Patra S., Behera C., Nayak R., Patil S., Bhutia S.K., Jena M. (2020). *Enteromorpha compressa* extract induces anticancer activity through apoptosis and autophagy in oral cancer. Mol. Biol. Rep..

[33] Pradhan B., Patra S., Behera C., Nayak R., Jit B.P., Ragusa A., Jena M. (2021). Preliminary Investigation of the Antioxidant, Anti-Diabetic, and Anti-Inflammatory Activity of *Enteromorpha intestinalis* Extracts. Molecules.

[34] Mohanty S., Pradhan B., Patra S., Behera C., Nayak R., Jena M. (2020). Screening for nutritive bioactive compounds in some algal strains isolated from coastal Odisha. J. Adv. Plant Sci..

[35] Singh S., Kola P., Kaur D., Singla G., Mishra V., Panesar P.S., Mallikarjunan K., Krishania M. (2021). Therapeutic Potential of Nutraceuticals and Dietary Supplements in the Prevention of Viral Diseases: A Review. Front. Nutr..

[36] Buck C.B., Thompson C.D., Roberts J.N., Müller M., Lowy D.R., Schiller J.T. (2006). Carrageenan is a potent inhibitor of papillomavirus infection. PLoS Pathog..

[37] Li M., Shang Q., Li G., Wang X., Yu G. (2017). Degradation of Marine Algae-Derived Carbohydrates by Bacteroidetes Isolated from Human Gut Microbiota. Mar. Drugs.

[38] Grassauer A., Weinmuellner R., Meier C., Pretsch A., Prieschl-Grassauer E., Unger H. (2008). Iota-Carrageenan is a potent inhibitor of rhinovirus infection. Virol. J..

[39] Hilliou L., Larotonda F.D., Abreu P., Ramos A.M., Sereno A.M., Gonçalves M.P. (2006). Effect of extraction parameters on the chemical structure and gel properties of kappa/iota-hybrid carrageenans obtained from *Mastocarpus stellatus*. Biomol. Eng..

[40] Koenighofer M., Lion T., Bodenteich A., Prieschl-Grassauer E., Grassauer A., Unger H., Mueller C.A., Fazekas T. (2014). Carrageenan nasal spray in virus confirmed common cold: Individual patient data analysis of two randomized controlled trials. Multidiscip. Respir. Med..

[41] Witvrouw M., Este J.A., Mateu M.Q., Reymen D., Andrei G., Snoeck R., Ikeda S., Pauwels R., Bianchini N.V., Desmyter J. (1994). Activity of a Sulfated Polysaccharide Extracted from the Red Seaweed *Aghardhiella Tenera* against Human Immunodeficiency Virus and Other Enveloped Viruses. Antivir. Chem. Chemother..

[42] Rodríguez M.C., Merino E.R., Pujol C.A., Damonte E.B., Cerezo A.S., Matulewicz M.C. (2005). Galactans from cystocarpic plants of the red seaweed *Callophyllis variegata* (Kallymeniaceae, Gigartinales). Carbohydr. Res..

[43] Matsuhiro B., Conte A.F., Damonte E.B., Kolender A.A., Matulewicz M.C., Mejías E.G., Pujol C.A., Zúñiga E.A. (2005). Structural analysis and antiviral activity of a sulfated galactan from the red seaweed *Schizymenia binderi* (Gigartinales, Rhodophyta). Carbohydr. Res..

[44] Queiroz K.C., Medeiros V.P., Queiroz L.S., Abreu L.R., Rocha H.A., Ferreira C.V., Jucá M.B., Aoyama H., Leite E.L. (2008). Inhibition of reverse transcriptase activity of HIV by polysaccharides of brown algae. Biomed. Pharmacother..

[45] McCandless E.L., Craigie J.S. (1979). Sulfated Polysaccharides in Red and Brown Algae. Annu. Rev. Plant Physiol..

[46] Akamatsu E., Shimanaga M., Kamei Y. (2003). Isolation of an anti-influenza virus substance, MC26 from a marine brown alga, *Sargassum piluliferum* and its antiviral activity against influenza virus. Coastal Bioenvironment.

[47] Hidari K.I., Takahashi N., Arihara M., Nagaoka M., Morita K., Suzuki T. (2008). Structure and anti-dengue virus activity of sulfated polysaccharide from a marine alga. Biochem. Biophys. Res. Commun..

[48] Hemmingson J.A., Falshaw R., Furneaux R., Thompson K. (2006). Structure and Antiviral Activity of the Galactofucan Sulfates Extracted from *Undaria Pinnatifida* (Phaeophyta). J. Appl. Phycol..

[49] Nelson T.E., Lewis B.A. (1974). Separation and characterization of the soluble and insoluble components of insoluble laminaran. Carbohydr. Res..

[50] Muto S., Niimura K., Oohara M., Oguchi Y., Matsunaga K., Hirose K., Kakuchi J., Sugita N., Furusho T., Yoshikumi C. (1992). Polysaccharides and antiviral drugs containing the same as active ingredient. U.S. Patent.

[51] Kanekiyo K., Hayashi K., Takenaka H., Lee J.B., Hayashi T. (2007). Anti-herpes simplex virus target of an acidic polysaccharide, nostoflan, from the edible blue-green alga *Nostoc flagelliforme*. Biol. Pharm. Bull..

[52] Lee J.B., Hayashi K., Hirata M., Kuroda E., Suzuki E., Kubo Y., Hayashi T. (2006). Antiviral sulfated polysaccharide from Navicula directa, a diatom collected from deep-sea water in Toyama Bay. Biol. Pharm. Bull..

[53] Hasui M., Matsuda M., Okutani K., Shigeta S. (1995). In vitro antiviral activities of sulfated polysaccharides from a marine microalga (*Cochlodinium polykrikoides*) against human immunodeficiency virus and other enveloped viruses. Int. J. Biol. Macromol..

[54] Yim J.H., Kim S.J., Ahn S.H., Lee C.K., Rhie K.T., Lee H.K. (2004). Antiviral Effects of Sulfated Exopolysaccharide from the Marine Microalga *Gyrodinium impudicum* Strain KG03. Mar. Biotechnol..

[55] Chen X., Han W., Wang G., Zhao X. (2020). Application prospect of polysaccharides in the development of anti-novel coronavirus drugs and vaccines. Int. J. Biol. Macromol..

[56] Pérez-Riverol A., Piñón R.A., Morier D.L.F., Torres L.Y., Mendoza L.D., del Barrio A.G. (2014). Antiviral activity of an aqueous extract from the red alga *Laurencia obtusa* against influenza A and B viruses. Rev. Cubana Med. Trop..

[57] Shih S.R., Tsai K.N., Li Y.S., Chueh C.C., Chan E.C. (2003). Inhibition of enterovirus 71-induced apoptosis by allophycocyanin isolated from a blue-green alga *Spirulina platensis*. J. Med. Virol..

[58] Soares A.R., Robaina M., Mendes G.S., Silva T.S.L., Gestinari L., Pamplona O.S., Yoneshigue-Valentin Y., Kaiser C.R., Romanos M.T.V. (2012). Antiviral activity of extracts from Brazilian seaweeds against herpes simplex virus. Rev. Bras. Farmacogn..

[59] Zaid S.A.A.-L., Hamed N.N.E.-D., Abdel-Wahab K.S.E.-D., Abo El-Magd E.K., Salah El-Din R.A.-L. (2016). Antiviral activities and phytochemical constituents of Egyptian marine seaweeds (*CystoseiraMyrica*(SG Gmelin) C. Agardh and *Ulva Lactuca* Linnaeus) aqueous extract. Egypt. J. Hosp. Med..

[60] Ohta S., Ono F., Shiomi Y., Nakao T., Aozasa O., Nagate T., Kitamura K., Yamaguchi S., Nishi M., Miyata H. (1998). Anti-Herpes Simplex Virus substances produced by the marine green alga, *Dunaliella primolecta*. J. Appl. Phycol..

[61] Wijesekara I., Yoon N.Y., Kim S.K. (2010). Phlorotannins from *Ecklonia cava* (Phaeophyceae): Biological activities and potential health benefits. Biofactors.

[62] Hayashi T., Hayashi K., Maeda M., Kojima I. (1996). Calcium spirulan, an inhibitor of enveloped virus replication, from a blue-green alga *Spirulina platensis*. J. Nat. Prod..

[63] Cardozo F.T., Larsen I.V., Carballo E.V., Jose G., Stern R.A., Brummel R.C., Camelini C.M., Rossi M.J., Simões C.M., Brandt C.R. (2013). In Vivo Anti-Herpes Simplex Virus Activity of a Sulfated Derivative of *Agaricus brasiliensis* Mycelial Polysaccharide. Antimicrob. Agents Chemother..

[64] Ray B., Ali I., Jana S., Mukherjee S., Pal S., Ray S., Schütz M., Marschall M. (2022). Antiviral Strategies Using Natural Source-Derived Sulfated Polysaccharides in the Light of the COVID-19 Pandemic and Major Human Pathogenic Viruses. Viruses.

[65] Zeitlin L., Whaley K.J., Hegarty T.A., Moench T.R., Cone R.A. (1997). Tests of vaginal microbicides in the mouse genital herpes model. Contraception.

[66] Xin X., Ding H., Geng M., Liang P., Li Y., Guan H. (2000). Studies of the anti-AIDS effects of marine polysaccharide drug 911 and its related mechanisms of action. Chin. J. Mar. Drugs.

[67] Xin X., Geng M., Guan H., Li Z. (2000). Study on the mechanism of inhibitory action of 911 on replication of HIV-1 in vitro. Chin. J. Mar. Drugs.

[68] Singh R.S., Walia A.K. (2018). Lectins from red algae and their biomedical potential. J. Appl. Phycol..

[69] Ingale A.G., Hivrale A.U. (2013). Plant as a plenteous reserve of lectin. Plant Signal. Behav..

[70] Mori T., Boyd M.R. (2001). Cyanovirin-N, a potent human immunodeficiency virus-inactivating protein, blocks both CD4-dependent and CD4-independent binding of soluble gp120 (sgp120) to target cells, inhibits sCD4-induced binding of sgp120 to cell-associated CXCR4, and dissociates bound sgp120 from target cells. Antimicrob. Agents Chemother..

[71] Shahzad-ul-Hussan S., Gustchina E., Ghirlando R., Clore G.M., Bewley C.A. (2011). Solution Structure of the Monovalent Lectin Microvirin in Complex with Manα(1–2)Man Provides a Basis for Anti-HIV Activity with Low Toxicity. J. Biol. Chem..

[72] Mori T., O’Keefe B.R., Sowder R.C., Bringans S., Gardella R., Berg S., Cochran P., Turpin J.A., Buckheit R.W., McMahon J.B. (2005). Isolation and characterization of griffithsin, a novel HIV-inactivating protein, from the red alga *Griffithsia* sp.. J. Biol. Chem..

[73] Meuleman P., Albecka A., Belouzard S., Vercauteren K., Verhoye L., Wychowski C., Leroux-Roels G., Palmer K.E., Dubuisson J. (2011). Griffithsin has antiviral activity against hepatitis C virus. Antimicrob. Agents Chemother..

[74] Takebe Y., Saucedo C.J., Lund G., Uenishi R., Hase S., Tsuchiura T., Kneteman N., Ramessar K., Tyrrell D.L., Shirakura M. (2013). Antiviral lectins from red and blue-green algae show potent in vitro and in vivo activity against hepatitis C virus. PLoS ONE.

[75] Nixon B., Stefanidou M., Mesquita P.M., Fakioglu E., Segarra T., Rohan L., Halford W., Palmer K.E., Herold B.C. (2013). Griffithsin protects mice from genital herpes by preventing cell-to-cell spread. J. Virol..

[76] O’Keefe B.R., Giomarelli B., Barnard D.L., Shenoy S.R., Chan P.K., McMahon J.B., Palmer K.E., Barnett B.W., Meyerholz D.K., Wohlford-Lenane C.L. (2010). Broad-spectrum in vitro activity and in vivo efficacy of the antiviral protein griffithsin against emerging viruses of the family Coronaviridae. J. Virol..

[77] Millet J.K., Séron K., Labitt R.N., Danneels A., Palmer K.E., Whittaker G.R., Dubuisson J., Belouzard S. (2016). Middle East respiratory syndrome coronavirus infection is inhibited by griffithsin. Antiviral Res..

[78] Bokesch H.R., O’Keefe B.R., McKee T.C., Pannell L.K., Patterson G.M., Gardella R.S., Sowder R.C., Turpin J., Watson K., Buckheit R.W. (2003). A potent novel anti-HIV protein from the cultured cyanobacterium *Scytonema varium*. Biochemistry.

[79] Li Y., Zhang X., Chen G., Wei D., Chen F. (2008). Algal lectins for potential prevention of HIV transmission. Curr. Med. Chem..

[80] Garrison A.R., Giomarelli B.G., Lear-Rooney C.M., Saucedo C.J., Yellayi S., Krumpe L.R., Rose M., Paragas J., Bray M., Olinger G.G. (2014). The cyanobacterial lectin scytovirin displays potent *in vitro* and *in vivo* activity against Zaire Ebola virus. Antiviral Res..

[81] Guidotti L.G., Chisari F.V. (1996). To kill or to cure: Options in host defense against viral infection. Curr. Opin. Immunol..

[82] Rajarshi K., Khan R., Singh M.K., Ranjan T., Ray S., Ray S. (2021). Essential functional molecules associated with SARS-CoV-2 infection: Potential therapeutic targets for COVID-19. Gene.

[83] Ramus J., Loewus F. (1973). Cell surface polysaccharides of the red alga *Porphyridium*. Biogenesis of Plant Cell Wall Polysaccharides.

[84] Nagle V., Gaikwad M., Pawar Y., Dasgupta S. (2020). Marine Red Alga *Porphyridium* sp. as a Source of Sulfated Polysaccharides (SPs) for Combating Against COVID-19. Preprints.

[85] Pereira L., Critchley A.T. (2020). The COVID 19 novel coronavirus pandemic 2020: Seaweeds to the rescue? Why does substantial, supporting research about the antiviral properties of seaweed polysaccharides seem to go unrecognized by the pharmaceutical community in these desperate times?. J. Appl. Phycol..

[86] Safarzadeh M., Sadeghi S., Azizi M., Rastegari-Pouyani M., Pouriran R., Hoseini M.H.M. (2021). Chitin and chitosan as tools to combat COVID-19: A triple approach. Int. J. Biol. Macromol..

[87] Koehn F.E., Sarath G.P., Neil D.N., Cross S.S. (1991). Halitunal, an unusual diterpene aldehyde from the marine alga *Halimeda tuna*. Tetrahedron Lett..

[88] Petit L., Vernès L., Cadoret J.P. (2021). Docking and *in silico* toxicity assessment of *Arthrospira* compounds as potential antiviral agents against SARS-CoV. J. Appl. Phycol..

[89] Alam M.A., Parra-Saldivar R., Bilal M., Afroze C.A., Ahmed M.N., Iqbal H.M.N., Xu J. (2021). Algae-Derived Bioactive Molecules for the Potential Treatment of SARS-CoV. Molecules.

[90] Chen C.Z., Shinn P., Itkin Z., Eastman R.T., Bostwick R., Rasmussen L., Huang R., Shen M., Hu X., Wilson K.M. (2020). Drug Repurposing Screen for Compounds Inhibiting the Cytopathic Effect of SARS-CoV. Front. Pharmacol..

[91] Talukdar J., Dasgupta S., Nagle V., Bhadra B. (2020). COVID-19: Potential of Microalgae Derived Natural Astaxanthin As Adjunctive Supplement in Alleviating Cytokine Storm. SSRN Electron. J..

[92] Park J.Y., Kim J.H., Kwon J.M., Kwon H.J., Jeong H.J., Kim Y.M., Kim D., Lee W.S., Ryu Y.B. (2013). Dieckol, a SARS-CoV 3CL^pro^ inhibitor, isolated from the edible brown algae *Ecklonia cava*. Bioorg. Med. Chem..

[93] Zumla A., Chan J.F., Azhar E.I., Hui D.S., Yuen K.Y. (2016). Coronaviruses—Drug discovery and therapeutic options. Nat. Rev. Drug Discov..

[94] Hirahashi T., Matsumoto M., Hazeki K., Saeki Y., Ui M., Seya T. (2002). Activation of the human innate immune system by Spirulina: Augmentation of interferon production and NK cytotoxicity by oral administration of hot water extract of *Spirulina platensis*. Int. Immunopharmacol..

[95] Wu Q., Liu L., Miron A., Klímová B., Wan D., Kuča K. (2016). The antioxidant, immunomodulatory, and anti-inflammatory activities of Spirulina: An overview. Arch. Toxicol..

[96] Chei S., Oh H.J., Song J.H., Seo Y.J., Lee K., Kim K.J., Lee B.Y. (2020). *Spirulina maxima* extract prevents activation of the NLRP3 inflammasome by inhibiting ERK signaling. Sci. Rep..

[97] Furukawa S., Kawabe H., Ohori H., Mukai T., Matsumoto M. (2008). Preventive or therapeutic composition for viral infectious disease. U.S. Patent.

[98] De Mello M.T., Silva A., de Carvalho Guerreiro R., Da-Silva F.R., Esteves A.M., Poyares D., Piovezan R., Treptow E., Starling M., Rosa D.S. (2020). Sleep and COVID-19: Considerations about immunity, pathophysiology, and treatment. Sleep Sci..

[99] Heo S.Y., Ko S.C., Kim C.S., Oh G.W., Ryu B., Qian Z.J., Kim G., Park W.S., Choi I.W., Phan T.T. (2017). A heptameric peptide purified from *Spirulina* sp. gastrointestinal hydrolysate inhibits angiotensin I-converting enzyme- and angiotensin II-induced vascular dysfunction in human endothelial cells. Int. J. Mol. Med..

[100] Zuo T., Zhang F., Lui G.C.Y., Yeoh Y.K., Li A.Y.L., Zhan H., Wan Y., Chung A.C.K., Cheung C.P., Chen N. (2020). Alterations in Gut Microbiota of Patients With COVID-19 During Time of Hospitalization. Gastroenterology.

[101] He Y., Wang J., Li F., Shi Y. (2020). Main Clinical Features of COVID-19 and Potential Prognostic and Therapeutic Value of the Microbiota in SARS-CoV-2 Infections. Front. Microbiol..

[102] Fields F.J., Lejzerowicz F., Schroeder D., Ngoi S.M., Tran M., McDonald D., Jiang L., Chang J.T., Knight R., Mayfield S. (2020). Effects of the microalgae *Chlamydomonas* on gastrointestinal health. J. Funct. Foods.

[103] Neyrinck A.M., Taminiau B., Walgrave H., Daube G., Cani P.D., Bindels L.B., Delzenne N.M. (2017). Spirulina Protects against Hepatic Inflammation in Aging: An Effect Related to the Modulation of the Gut Microbiota?. Nutrients.

[104] Chandrarathna H., Liyanage T.D., Edirisinghe S.L., Dananjaya S.H.S., Thulshan E.H.T., Nikapitiya C., Oh C., Kang D.H., De Zoysa M. (2020). Marine Microalgae, *Spirulina maxima*-Derived Modified Pectin and Modified Pectin Nanoparticles Modulate the Gut Microbiota and Trigger Immune Responses in Mice. Mar. Drugs.

[105] Kim K., Ehrlich A., Perng V., Chase J.A., Raybould H., Li X., Atwill E.R., Whelan R., Sokale A., Liu Y. (2019). Algae-derived β-glucan enhanced gut health and immune responses of weaned pigs experimentally infected with a pathogenic *E. coli*. Anim. Feed Sci. Technol..

[106] Chen L., Xu W., Chen D., Chen G., Liu J., Zeng X., Shao R., Zhu H. (2018). Digestibility of sulfated polysaccharide from the brown seaweed *Ascophyllum nodosum* and its effect on the human gut microbiota in vitro. Int. J. Biol. Macromol..

[107] Rodrigues D., Walton G., Sousa S., Rocha-Santos T.A.P., Duarte A.C., Freitas A.C., Gomes A.M.P. (2016). *In vitro* fermentation and prebiotic potential of selected extracts from seaweeds and mushrooms. LWT.

[108] Delgado-Roche L., Mesta F. (2020). Oxidative Stress as Key Player in Severe Acute Respiratory Syndrome Coronavirus (SARS-CoV) Infection. Arch. Med. Res..

